# Medical Department of the University of Buffalo

**Published:** 1853-04

**Authors:** 


*Medical Department of the University of Buffalo.* — The annual commencement in this institution will be on the 27th inst. The commencement exercises will consist of the conferring of degrees upon the successful candidates, accompanied by addresses, on the part of the Council of the University, by His Honor, George W. Clinton, President of the Council, and, on the part of the Medical Faculty, by Prof. Frank H. Hamilton. These exercises will take place in the evening of the 27th instant. Due notice of the place and hour will be given in the daily city papers.

The examination of candidates before the curators of the medical department of the University, will take place at the College building, on the 27th inst., to commence at ten o'clock, A. M. The curators will please notice this announcement, and they are respectfully invited to attend without more formal invitation.

The attendance during the course of lectures now drawing to a close has been, in numbers, respectable, and in the character of the class, aside from magnitude, more than respectable. For attention, zeal, and orderly deportment, the students assembled at this institution during the past winter, have been particularly distinguished.

The connection of Dr. E. M. Moore, of Rochester, N. Y., with this school, having commenced with the present session, we may with propriety advert to the impression which he has produced as a teacher of anatomy. His success has in every respect justified the expectations of his friends, and realized all that could be desired by the friends of the school. Thoroughly versed in his department of instruction, and especially familiar with its important practical relations, he possesses, in an admirable degree, the sterling qualities of a sound and instructive lecturer, viz., method, clearness, fluency, a chaste style, and an agreeable manner. The institution may well be congratulated that this important chair is so satisfactorily filled.

The permanent appointmeut of Prof. Dalton dated with the beginning of the present collegiate year. Our columns have borne testimony to the residence of Dr. D. in Europe during the last summer and autumn, for the purpose of prosecuting, under the most favorable circumstances, studies in physiology and morbid anatomy; and his lectures during the present session, especially in the former of these branches, have afforded abundant evidence of the extent and accuracy of his attainments in the recent developments due to organic chemistry, the microscope, and experimental researches. It is but simple justice to say that the lectures on physiology, with the accompanying microscopical demonstrations, and illustrations by experiments on living animals, have excited among the class a degree of enthusiasm which will become the basis of a lasting interest in that most attractive, progressive, and important province of medical science.

Of the two members of the Faculty just referred to, it does not seem to us indecorous thus to speak, inasmuch as they have recently become connected with the University. This fact, while it suffices for an explanation (if any be required) will also divest our remarks of any invidiousness which might otherwise attach to them.

